# Fecal microbiota transplantation for patients on antibiotic treatment with *C. difficile* infection history (GRAFT): Study protocol for a phase II, randomized, double-blind, placebo-controlled trial to prevent recurrent *C. difficile* infections

**DOI:** 10.1016/j.conctc.2020.100576

**Published:** 2020-05-19

**Authors:** Ashley E. Kates, Ilsa Gaulke, Travis De Wolfe, Michele Zimbric, Kendra Haight, Lauren Watson, Garret Suen, Kyungmann Kim, Nasia Safdar

**Affiliations:** a Division of Infectious Disease, Department of Medicine, School of Medicine and Public Health, University of Wisconsin-Madison, Madison, WI, USA; b William S. Middleton Veterans Affairs Medical Center, Madison, WI, USA; d Department of Bacteriology, College of Agricultural and Life Sciences, University of Wisconsin-Madison, Madison, WI, USA; e Department of Biostatistics & Medical Informatics, School of Medicine and Public Health, University of Wisconsin-Madison, Madison, WI, USA

**Keywords:** Clostridioides difficile, Microbiome, 16S rRNA sequencing

## Abstract

Recurrent *Clostridiodes difficile* infections (rCDIs) are a burdensome problem. Patients with a history of CDI that are prescribed antibiotics are at a high risk for recurrence. Fecal microbiota transplantation (FMT) has been shown to be an effective treatment for rCDI, though there is little information on the impact of FMT with antibiotics on the gut microbiome. We are conducting a clinical trial of FMT to prevent rCDI in patients with a history of CDI currently taking antibiotics. Our primary objective is to determine the effect of FMT on the gut microbiome during antibiotic exposure. Our secondary aim is to assess safety and feasibility of using FMT as a prophylaxis for CDI. We plan to enroll 30 patients into a phase II randomized, double-blind, placebo-controlled trial with three arms: (1) 5 FMT capsules per day during antibiotic treatment and for 7 days post antibiotic cessation, (2) a one-time dose of 30 FMT capsules 48–72 h post cessation of antibiotic treatment, or (3) 5 placebo capsules per day during antibiotic treatment and for 7 days post antibiotic treatment. Patients provide stool samples throughout the duration of the study and are cultured *C. difficile.* Sequencing of the V4 region of the 16S rRNA gene will be carried out to assess the gut microbiota. Results of this study will provide information on the impact of FMT on the gut microbiome as well as the necessary data to examine whether or not prophylactic FMT should be explored further as a way to prevent CDI recurrence.

## 1 Introduction

*Clostridioides difficile* (formally *Clostridium difficile)* infection (CDI) is considered an urgent public health threat by the Centers for Disease Control and Prevention [1] and was the number one pathogen causing healthcare-associated infections in 2015 [2]. Recurrence rates for CDIs have been reported to occur in an average of 20% of primary cases due to increased treatment difficulty [[3], [4], [5]]. Furthermore, one repeated recurrence is followed by additional repeated episodes in 65% of patients [6].

Recurrent CDI is thought to be due to the inability of the intestinal microbiota to re-establish after antibiotic treatment causing a state of dysbiosis. This dysbiosis leaves the patient susceptible to infection either by a new strain of *C. difficile* or re-infection with the original infecting strain [7]. New antibiotic exposure is one of the major risk factors for recurrent CDIs [6].

Fecal microbiota transplantation (FMT) therapy is the process of restoring a healthy gut microbial composition via the transfer of feces from a healthy donor to the gut of the patient in a state of gastrointestinal microbial dysbiosis [8]. In patients with recurrent CDI, the gut microbiota are in a continuous state of dysbiosis allowing *C. difficile* to proliferate [8]. FMT has been shown to be a highly effective and inexpensive treatment for recurrent CDI with studies showing up to 89% of patients treated with FMT having resolution of their CDI after just one treatment and limited side effects [7,9]. Currently FMT is recommended for patients with multiple recurrent CDI, moderate CDI not responding to standard therapy of vancomycin or fidaxomicin for 1 week, and severe or fulminant CDI not responding to standard therapy after 48 h [10].

Here we describe the study protocol for a phase II randomized, double-blind, placebo-controlled trial to assess oral FMT therapy and its impact on the gut microbiome. We will evaluate the effect of two different methods of FMT administration – one administered at a single, high dose following cessation of antibiotic treatment or one administered at a low dose daily during antibiotic treatment and for one week following antibiotic cessation – compared to placebo, and their impact on the gastrointestinal microbiome. Given the high rate of recurrent CDI infections and the safety and efficacy of FMT to return the gut microbiome to a state of symbiosis, FMT as prevention for CDI could be a cost-effective way to reduce the burden of recurrent CDI.

## 2 Methods

### 2.1 Study design, aims, and hypotheses

#### 2.1.1 Study design

**Fig. 1:**
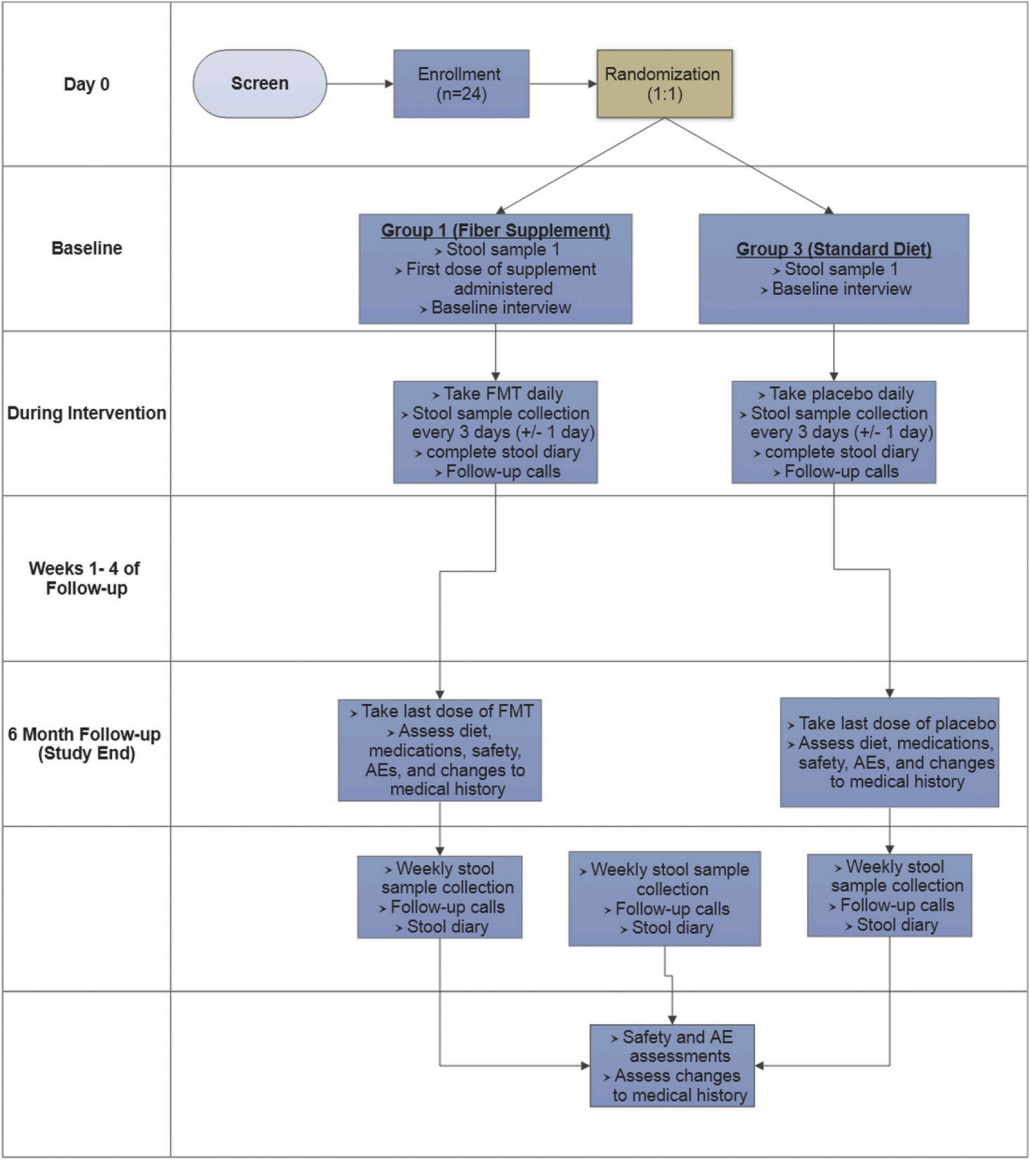
Schematic depiction of the GRAFT study procedures. AE: adverse event.

This is a phase II, randomized, double-blind, placebo-controlled trial of adult patients with a history of CDI who have been recently prescribed antibiotics for infections other than *C. difficile.* Patients are placed into one of three treatment groups: (1) a low dose of 5 FMT capsules per day during antibiotic treatment and for 7 days post antibiotic cessation, (2) a one-time dose (high dose) of 30 FMT capsules 48–72 h post cessation of antibiotic treatment, or (3) a low dose of 5 placebo capsules per day during antibiotic treatment and for 7 days post antibiotic cessation. Patients are randomized in a 1:1:1 ratio using permuted blocks in sizes of 3 and 6. Fig. 1 provides an overview of the design and methods.

#### 2.1.2 Study population

We plan to enroll 30 patients into this clinical trial (10 per group). Children under 18 years of age are not included due to uncertainty of the safety of the intervention. Participants must meet all inclusion criteria and have no exclusion criteria to be enrolled (Table 1, Table 2). Patients are enrolled at a large, academic teaching hospital in Wisconsin. We expect the study population to be predominantly Caucasian due to the demographics of the region, with approximately half the subjects being female. Both inpatients and outpatients are included in this trial. All participants must have had a CDI within the last 180 days, be taking an antibiotic for a reason other than CDI, and are identified via a medical record review.

**Table 1:** Inclusion criteria for enrollment in the trial.

Cognitively intact and willing to provide informed consent
Willing and able to comply with all study procedures for the duration of the study
Able to take oral medications
At least 18 years of age
CDI within the last 180 days with completion of therapy as documented in the electronic medical record
Receiving antibiotics at enrollment for reasons other than CDI and having taken the antibiotics for no longer than 72 h at enrollment
Women of childbearing potential in a sexual relationship with men must use an acceptable method of contraception (including, but not limited to, barriers with additional spermicidal foam or jelly, intrauterine devices, hormonal contraception started at least 30 days before enrollment into the study, or intercourse with men who underwent a vasectomy) for 4 weeks following completion of the study treatment.
Males must agree to avoid impregnation of women during and for 4 weeks following completion of the study treatment
Able to take the test capsule successfully with no signs of dysphagia

**Table 2:** Exclusion criteria for enrollment in the trial.

Admitted to an intensive care unit
Females who are pregnant, lactating, or planning to become pregnant during the study. Female patients of childbearing potential will take a pregnancy test at the intervention visit and will be excluded if pregnant
Inability (e.g. dysphasia) to or unwilling to swallow capsules
Known or suspected toxic megacolon and/or small bowel ileus
Bowel obstruction or other gut motility issues occurring in the last two weeks that are unresolved as noted by the patient or in the EMR.
Major gastrointestinal surgery (e.g. significant bowel resection) within 3 months before enrollment not including appendectomy or cholecystectomy.
History of bariatric or total colectomy surgery
Current intensive induction chemotherapy, radiation therapy, or biologic treatment for an active malignancy. Patients on maintenance chemotherapy may be enrolled after consultation with the medical monitor.
Expected life expectancy less than 6 months.
Patients with severe anaphylactic or anaphylactoid food allergy.
Solid organ transplant recipients less than or equal to 90 days post-transplant or on active treatment for rejection.
Neutropenia (≤500 neutrophils/mL) or other severe immunosuppression. Anti-TNF will be permitted. Patients on monoclonal antibodies to B and T cells, glucocorticoids, antimetabolites (azathioprine, 6-mercaptoputine, methotrexate), calcineurin inhibitors (tacrolimus, cyclosporine), and mycophenolate mofetil may only be enrolled after consultation with the medical monitor.
At risk of cytomegalovirus (CMV) or Epstein Barr virus (EBV) associated disease (negative IgG testing).
Any other infectious gastrointestinal illness including diarrhea.
On oral vancomycin or metronidazole.
Having been taking the currently prescribed antibiotic longer than 72 h.
On an antibiotic treatment anticipated to exceed 19 days.
Having received FMT by any route in the 180 days prior to enrollment.
Any condition that would jeopardize the safety or rights of the patient, would make it unlikely for the patient to complete the study, or would confound the results of the study.

#### 2.1.3 Aims and hypotheses

There are two main objectives to this clinical trial. The first is to determine the effects of FMT therapy compared to placebo on the composition and function of the gut microbiome in patients with recent CDI who are currently taking antibiotics. We hypothesize FMT therapy will maintain the diversity of the gut microbiota and therefore reduce the risk of recurrent CDI. The second is to assess the feasibility and safety of concurrent oral FMT and antibiotic treatment in high-risk patients, with the hypothesis that oral FMT treatment will be safe.

To test our hypotheses, we will collect serial stool samples from patients every 3 days starting after randomization and continuing until the intervention dosing is complete. We will then collect weekly stool samples for four weeks following the completion of study product administration. From these samples we will culture for *C. difficile* as well as other multidrug resistant organisms (MDROs) and extract and sequence total genomic DNA. We will compare alpha and beta diversity indices across each timepoint and across patient groups. We will also conduct weekly calls with patients to assess safety and protocol adherence up to 6 months post study completion.

### 2.2 Recruitment and consent

Inpatients and outpatients are identified via daily generated lists for all patients in our health system who have a positive *C. difficile* test and through automatic, real-time electronic medical record (EMR) notification to study coordinators of patients with a positive *C. difficile* test in the last 180 days who have antibiotics initiated. Inpatients are also identified through a list generated by the Infection Control team listing all patients admitted to the hospital for an infectious disease.

Study coordinators and medical monitor review the patients’ EMR to determine eligibility prior to approaching the patient. If the patient is potentially eligible, the study team visits the inpatient to discuss the study, determine interest, and answer any questions the patient may have.

Patients may also be enrolled from ambulatory settings. Outpatients known to have had a CDI in the last 180 days are mailed a letter describing the study and inviting them to participate should they be prescribed antibiotics in the future. The study team follows this letter with up to three phone calls to discuss the study with the patient to determine interest and eligibility. If the patient is interested, they are invited to contact the research team who then monitors the patient's EMR for antibiotic use. If/when the patient is prescribed antibiotics, they are screened for eligibility criteria and scheduled for an enrollment visit.

Outpatients identified through screening may also be contacted by a “cold call” to assess interest in the study if a patient is known to have been prescribed an antibiotic within the last 72 h, since a mailed letter would not reach them in time to remain eligible. Patients contacted by mail and/or phone who are interested in participating in the study and meet the inclusion criteria will be invited to a clinic visit for further screening and study enrollment. Clinic providers who have been given information on the study may introduce the study to their patients. Interested patients are encouraged to contact the study team and set an enrollment visit if interested.

At the enrollment visit a member of the research team will obtain written informed consent from the subject. Participants are paid $50.00 after completion of or termination from the study. All study documents and procedures have been approved by the University of Wisconsin-Madison Health Sciences Institutional Review Board (IRB ID: 2017–0789).

### 2.3 Intervention

The intervention is the administration of a double encapsulated (DE) FMT capsule. The capsule is a DE formulation of frozen fecal microbiota sourced from human-derived microbes. The capsule is designed for targeted delivery of the FMT product into the colon. The intervention consists of administering the FMT or placebo product either with a single dose of 30 FMT capsules within 48 h of completing antibiotics (single dose group) or consuming 5 capsules daily while on antibiotics and for 7 days after completing the antibiotic course (daily dose FMT and placebo groups). The placebo consists of the same inert filler ingredients as the FMT capsules and is identical to the active product in both appearance and taste. Both the FMT and placebo capsules are manufactured by Finch Therapeutics (Somerville, MA, USA) and distributed by OpenBiome (Cambridge, MA, USA).

### 2.4 Enrollment and study visits

#### 2.4.1 Baseline screening and enrollment visit

All patients with a history of CDI and on antibiotics who meet the eligibility criteria are eligible for enrollment. Eligibility is confirmed at the enrollment visit. A thorough consenting process is conducted with the patient by trained study staff prior to any other study procedures. Following consent, patients who meet eligibility criteria undergo a detailed clinical assessment where vital signs, demographics, medical history, dietary information, and current medications are collected. Data collection occurs through patient interviews and medical record review. Immunocompromised patients at risk for CMV/EBV have their blood drawn during the enrollment visit and CMV and EBV antibody tests done by the hospital's clinical lab.

For all patients, a baseline stool sample or perirectal swabs are collected. After the sample is collected and all pre-dose procedures are complete, the patient is randomized to one of the three intervention aims. Prior to administering the intervention, patients are asked to swallow a test capsule to confirm they are able to swallow pills without dysphagia. If the patient is in the daily FMT or placebo group and is able to swallow the test capsule without problems, they can be given the first dose of the intervention treatment. The intervention is administered by the blinded treatment staff. Following the intervention, the patient is observed for 30 min post-ingestion for adverse events. Patients in the daily groups are placed on a clear liquid only diet for 2 h prior to receiving the intervention each day and for 1 h post. All patients begin filling out stool diaries to track their bowel movements after the first visit. Patients are also given a symptom log to collect information on any symptoms they may experience. Patients in the daily groups track their study product intake on a medication log.

#### 2.4.2 Subject follow-up

Patients fill out their diaries daily and provide a stool sample every three days ( ±1 day) following the baseline visit until the intervention is completed. Once the intervention is complete, patients are followed for an additional four weeks providing weekly stool samples. Outpatients and patients discharged during the follow-up period are called or emailed (depending on patient preference) weekly to remind them to take their capsules and to send in their stool samples. The researcher calling the subject also asks the subject to count the number of pills he or she has remaining (recorded in the medication log) and ask about any adverse events or changes to medications and diet.

For inpatients in the daily intervention groups, capsules are delivered to the inpatient rooms every day by a blinded member of the treatment team during the time they are admitted. If the patient is discharged or an outpatient, they are provided with enough capsules to complete the study and are asked to consume 5 capsules per day while on antibiotics as well as for 7 days post-antibiotic treatment cessation.

For patients in the single-dose intervention group, the intervention is administered under the supervision of the research team 48–72 h after completing the antibiotic regimen. Patients are asked to be on a clear liquid diet for 8 h prior to delivering the intervention. Prior to administration, a physical exam is conducted and vitals are collected to ensure the patient is able to undergo the intervention. The patient then consumes a test capsule just as is done for the daily-dose groups. If the patient is able to swallow the capsule, they then consume 30 capsules under the supervision of a study nurse or physician over a maximum of 90 min. As is done in the daily group, the patients are observed for 30 min for adverse events and allowed to resume a normal diet 1 h post-consumption.

#### 2.4.3 Unscheduled visits

An unscheduled visit is conducted when a patient develops a CDI or related serious adverse events (SAE) occur. The development of a CDI will result in early termination from the study. SAEs are evaluated on a case-by-case basis to determine if early termination is necessary. Unscheduled visits also occur when a patient develops a new infection. Any changes to the type of antibiotic(s), duration, and dosage will be recorded.

#### 2.4.4 Early termination

All subjects are informed during consent that they may discontinue participation at any time. If a participant wishes to withdraw, we ask the participant to schedule a close-out study visit to collect the final stool sample and safety evaluation. Additionally, a patient may be withdrawn from the study by the investigator if the patient experiences an adverse event making it no longer in the patient's best interest to continue participation, if the patient develops CDI, if the patient becomes pregnant, is non-compliant, or is lost to follow-up. In the event a patient is withdrawn from the study by the investigators, the primary reason for withdrawing is recorded and patients are asked to complete the early termination visit. During this visit a final stool sample is collected and safety evaluation is completed. If the patient is withdrawn due to an adverse event (AE) or SAE, the investigators will attempt to follow the patient by monitoring the medical record if the patient allows, until the event is resolved or stabilized. If a patient provided at least one stool sample prior to withdrawing, full compensation is provided.

#### 2.4.5 Study end

All patients are called 6 months ( ±2 weeks) following the completion of the intervention. During this call, the researcher asks the patient about any changes to medical history, any changes to their stool frequency and/or consistency following the Bristol stool scale, and any AEs that may have occurred since the last contact.

#### 2.4.6 Outcomes

The primary outcome is the microbial composition of the gut following the intervention and the changes to the gut's microbial composition during the intervention. The secondary outcomes are recurrence of CDI, colonization with *C. difficile,* time to CDI and/or colonization with *C. difficile* and safety and feasibility of the intervention. *C. difficile* presence will be considered a CDI and not colonization based on the presence of toxigenic *C. difficile* via polymerase chain reaction and the presence of clinical symptoms. Safety is defined as AEs, SAEs, and newly acquired transmissible infectious diseases (AEs of special interest) following randomization.

Data from case report forms will be stored in REDCap 8.1.1 electronic data capture system (ref). REDCap database enables secure user log-in for the research staff, data query rules, validated data entry, audit trails, and data resolution workflow features for managing and documenting data integrity and quality [11].

### 2.5 Safety

#### 2.5.1 FMT product and safety

OpenBiome's FMT Capsule DE is a double encapsulated system designed to deliver the product past the stomach. It contains fecal microbiota filtered to 330 μm, theobroma oil, glycerol, hide bovine gelatin, sodium lauryl sulfate, colorants, and titanium oxide. The product is stored between −20 °C and −80 °C and is considered expired 6 months after receipt. All donor stool undergoes strict screening for infectious and non-infectious pathogens that may be mediated by the gastrointestinal microbiome. Donors are screened by Finch Therapeutics using a detailed health questionnaire and laboratory testing of blood and stool prior to donation of feces, quarantining of the stool, and rescreening 60 days later. While no SAEs definitively related to OpenBiome's FMT product used have been reported, all FMT administrations comes with potential risk. Diarrhea, abdominal cramps or discomfort, nausea, fever, bloating, belching, vomiting, and constipation have been reported for the FMT product. It is also possible patients could develop infections, allergic reactions, and autoimmune conditions. There is also a theoretical risk of developing non-infectious diseases, such as obesity or metabolic syndrome, from the FMT product.

#### 2.5.2 Safety of placebo

The placebo consists of glycerol and saline. There is no foreseeable risk of adverse reactions to the placebo other than an allergic reaction, though safety assessment will be performed for unbiased assessment of the safety of the FMT product.

#### 2.5.3 Safety and data monitoring

All participants are instructed to contact the research team as well as their treating physician if they develop any signs of infection. The University of Wisconsin Institute for Clinical and Translational Research Data Monitoring Committee (UW-ICTR DMC) is the data and safety monitoring board of record for this trial. The UW-ICTR DMC reviews this study every six months over the duration of the trial to provide protocol oversight and assessment of AEs. In the event of any AEs, timely and accurate reporting and analysis of the safety information will be undertaken.

### 2.6 Sample collection

Stool samples are self-collected by the patient. If a patient is unable to provide a stool sample for a scheduled visit, perirectal swabs will be collected instead. Patients are provided with stool collection kits, shipping materials, and instructions for self-collecting stool and mailing it back to the research laboratory. Sample collection kits include a pair of clean gloves, a collection tub or “hat”, a wooden tongue depressor, biohazard bag, pre-filled specimen label, and a sterile specimen cup. No preservative agents are used. Participants are also provided insulated shipping containers and ice packs. Participants have a window of ± 1 day to collect the stool samples. To remind patients when to collect samples, a member of the study team performs weekly reminder calls and/or emails.

### 2.7 Laboratory analysis

When samples arrive at the research lab, they are processed for both molecular and microbiologic analyses using 16S rRNA sequencing and the detection of MDROs including but not limited to *C. difficile,* MRSA and VRE*.* For *C. difficile* culture, approximately 0.1–0.2 g of stool is inoculated into 1 mL of *C. difficile* Brucella broth and incubated anaerobically at 36 °C overnight. After incubation, 50 μL of the broth is plated to *C. difficile* Brucella agar and incubated again at 36 °C for 24–48 h [12]. Colonies matching the suspected colony morphology (yellow, irregular, ground glass colonies) are subcultured to a blood agar plate for 24 h for gram staining and catalase testing. Presence of house-keeping and toxin genes are assessed through a polymerase chain reaction (PCR) assay. To identify VRE, 0.1–0.2 g of stool is inoculated into Bile Esculin Azide (BEA) broth and vortexed for 15 s and incubated at 36 °C for 24 h. Fifty microliters are then plated to BEA agar with 6 μg/mL of vancomycin and incubated at 36 °C for 48 h. Suspected positive colonies are then plated to blood agar and incubated overnight at 36 °C aerobically for gram staining. Gram positive cocci testing catalase negative and pyrolidonyl arylamidase (PYR) positive will be considered Enterococcus and an E-test will be done to determine vancomycin resistance [13]. To identify MRSA, 0.1–0.2 g of stool is incubated for overnight at 36 °C in tryptic soy broth with sodium chloride before being plated to a mannitol salt agar plate supplemented with 4 mg/L cefoxitin and incubated for an additional 48 h at 36 °C. Suspected colonies are then plated to blood agar for gram staining, coagulase testing, and methicillin susceptibility testing [13,14].

For molecular analysis, 80–100 mg of stool is aliquoted into a bead beat tube containing three 1.0 mm glass beads for DNA extraction. Five hundred microliters of 2x sodium chloride-tris-EDTA (STE) buffer is added and vortexed to homogenize the stool and then centrifuged for 15 min at 4 °C at 500*g*. An additional 800 μL of STE buffer is added to the supernatant and up to 1000 μL is transferred to a new bead beat tube containing one 4 mm diameter stainless steel bead and 300 μL 0.1 mm zirconia/silica beads. For chemical lysis, 115 μL of an enzymatic cocktail is added to the sample (50 μL 10 mg/mL lysozyme, 10 μL 1 mg/mL mutanolysin, 5 μL 5 mg/mL lysostaphin, 50 μL 20% sodium dodecyl sulfate) with 700 μL phenol:chloroform:isoamyl alcohol. The bead beat tube is then incubated at 56 °C for 30 min. Tubes are then vortexed and bead beat for 3 min in a Mini-BeadBeater-24 (Cat 112,011, Biospec Products, Bartletsville, Oklahoma, USA). The tubes are then centrifuged at 16,000*g* for 10 min at 4 °C. The top aqueous layer is transferred to a new, sterile 2 mL tube and washed with an additional 500 μL phenol:chloroform:isoamyl alcohol, vortexed, and centrifuged again at 16,000*g* for 10 min at 4 °C. This will be repeated 2–10 times to remove impurities from the sample until the aqueous layer is cleared of debris. After the final wash, the top aqueous layer is then transferred to a clean microcentrifuge tube containing 70 μL of 3 M sodium acetate and 700 μL isopropanol. The samples are then inverted several times and incubated at −20 °C for 30 min to 1 h and centrifuged at 16,000*g* for 20 min at 4 °C to pellet the DNA. The pellet is then washed with 500 μL cold 70% ethanol. The ethanol wash is repeated and dried for 5 min using a Savant SpeedVac (DNA120-230, Thermo Scientific, Waltham, MA, USA). The dried pellet is resuspended in 100 μL TE buffer and stored overnight at 4 °C or at 37 °C for 1 h to dissolve the pellet. Samples are then purified using the NucleoSpin Gel and PCR clean-up kit according to manufacturer's directions (Macherey-Nagel, Germany) and eluted in 40 μL TE buffer. DNA is then quantified using PicoGreen in a microplate reader (BioTek Instruments, Winooski, VT, USA) and stored long term at −80 °C [14].

Following DNA extraction and quantitation,16S rRNA targeted amplicon sequencing of the V4 region will be performed on an Illumina MiSeq. All sequencing is carried out at the University of Wisconsin-Madison Biotechnology Center. DNA is normalized to 5 ng/μL and amplified using PCR with barcoded primers for the 16S rRNA V4 region and then sequenced on an Illumina MiSeq using a 2 × 250-bp paired-end MiSeq v2 sequencing kit (Illumina, San Diego, CA) [15].

### 2.8 Statistical analysis

16S rRNA sequences will be processed using VSEARCH [16]. Operational taxonomic units (OTUs) will be assigned taxonomy using the GreenGenes database [17] to the genus level whenever possible. Alpha diversity will be assessed using the Shannon [18] and Inverse Simpson's [19] diversity indices. The ACE and Chao1 indices will be used to assess richness [20]. Beta diversity will also be calculated to assess community structure.

Categorical data will be described using descriptive statistics (proportions and percentages). Continuous data will be described using means and standard deviations or medians and interquartile range. Appropriate comparative statistical tests will be chosen based in the variable types (categorical, dichotomous, continuous) and distribution and will be used to assess statistical significance of the difference between intervention and control groups. Covariates of interest include the biological variable of sex and past and current medications including antibiotics, dietary history, hospitalizations, reason for current antibiotics, stool consistency, current health and comorbidities, colonization with *C. difficile*, any adverse events or symptoms reported, as well as smoking and alcohol use. Where appropriate, point estimates of the treatment effect and associated 95%confidence intervals will be reported. All analyses will be conducted in R [21].

## 3 Discussion

Recurrent CDI is a burdensome and challenging problem and the use of antibiotics is a primary risk factor for recurrence. Treatment of recurrent CDI is less straightforward than primary CDI and multiple recurrences occur in approximately 50–65% of those patients who have had at least one recurrence [6]. In addition, patients hospitalized for CDIs are more ill than the general population and are at increased risk of death [22]. While the mechanism for recurrence is not fully understood, new or continued disruption to the gastrointestinal microbiota is known to reduce colonization resistance and put the patient at risk for recurrence [22]. Due to the high risk of recurrence when on antibiotics, preventing recurrent infection by reconstituting the gastrointestinal tract with microbes through FMT may reduce the risk of recurrence in high risk populations.

FMT has been used to successfully treat patients with recurrent CDI in numerous trials, although this is the first clinical trial we are aware of designed to use FMT as a prophylactic measure against CDI. This study is designed to begin to establish whether FMT is effective at reducing reinfections and recurrence as well as to understand patient safety and acceptability. This trial has the potential to reveal new insights into how the gut microbiota of those receiving antibiotics may be impacted by FMT therapy and if the therapeutic benefits of FMT are retained in the presence of concurrent antibiotic use.

While this study will help us to determine the safety and efficacy of FMT to prevent CDIs, it does have limitations. First, this is a small trial with a sample size goal of 10 patients per group. Although the sample size is small, one of the goals of this study is to determine feasibility and to determine if larger studies are warranted. Another potential limitation is that this is an involved study requiring intensive participation over several months, which may impact participation and adherence. To maximize participation and adherence, our research staff keep in close contact with the patients as well as collect information on a back-up contact person that can be reached if the patient becomes lost to follow-up. Additionally, the use of oral FMT capsules allows for patients in the daily group to receive therapy without having to return to the hospital or clinic. Lastly, the study team members administering the intervention will not be able to be fully blinded to treatment group as there is not placebo control for group 2. However, the analytic team will be blinded to intervention group.

New methods to prevent recurrent CDI is of importance for infection prevention as it would help reduce the reservoir of patients who are able to transmit *C. difficile* and could potentially reduce the number of patients on prolonged oral vancomycin, the current standard of care for recurrent CDI. After the completion of this trial, we hope to have provided new insights into the therapeutic benefits of FMT in high risk patients and to determine if prophylactic FMT is a treatment worth pursuing in larger trials. If FMT proves effective in this trial as a prophylactic against recurrent CDI, the knowledge gained may be applied to other preventative interventions, such as synthetic FMT or targeted probiotics, that could impact the gut by similar mechanisms.

## Funding

This project was supported by grant number R03HS025257 from the Agency for Healthcare Research and Quality and the Clinical and Translational Science Award (CTSA) program, through the NIH National Center for Advancing Translational Sciences (NCATS), grant UL1TR000427. The content is solely the responsibility of the authors and does not necessarily represent the official views of the Agency for Healthcare Research and Quality or the views of the NIH. AEK is supported by an 10.13039/100000092NLM training grant to the Computation and Informatics in Biology and Medicine Training Program (NLM5T15LM007359) and TJD is supported by the Pittsburgh Biomedical Informatics Training Program funded by the 10.13039/100000002National Institutes of Health (NLM5T15LM007059-32).
